# *Shewanella oneidensis* MR-1-Induced Fe(III) Reduction Facilitates Roxarsone Transformation

**DOI:** 10.1371/journal.pone.0154017

**Published:** 2016-04-21

**Authors:** Guowei Chen, Zhengchen Ke, Tengfang Liang, Li Liu, Gang Wang

**Affiliations:** 1 School of Civil and Hydraulic Engineering, Hefei University of Technology, Hefei 230009, China; 2 Department of Soil and Water Sciences, China Agricultural University, Beijing 100193, China; The University of Akron, UNITED STATES

## Abstract

Although microbial activity and associated iron (oxy)hydroxides are known in general to affect the environmental dynamics of 4-hydroxy-3-nitrobenzenearsonic acid (roxarsone), the mechanistic understanding of the underlying biophysico-chemical processes remains unclear due to limited experimental information. We studied how *Shewanella oneidensis* MR-1 –a widely distributed metal-reducing bacterium, in the presence of dissolved Fe(III), affects roxarsone transformations and biogeochemical cycling in a model aqueous system. The results showed that the MR-1 strain was able to anaerobically use roxarsone as a terminal electron acceptor and to convert it to a single product, 3-amino-4-hydroxybenzene arsonic acid (AHBAA). The presence of Fe(III) stimulated roxarsone transformation via MR-1-induced Fe(III) reduction, whereby the resulting Fe(II) acted as an efficient reductant for roxarsone transformation. In addition, the subsequent secondary Fe(III)/Fe(II) mineralization created conditions for adsorption of organoarsenic compounds to the yielded precipitates and thereby led to arsenic immobilization. The study provided direct evidence of *Shewanella oneidensis* MR-1-induced direct and Fe(II)-associated roxarsone transformation. Quantitative estimations revealed a candidate mechanism for the early-stage environmental dynamics of roxarsone in nature, which is essential for understanding the environmental dynamics of roxarsone and successful risk assessment.

## Introduction

**Fig 1:**
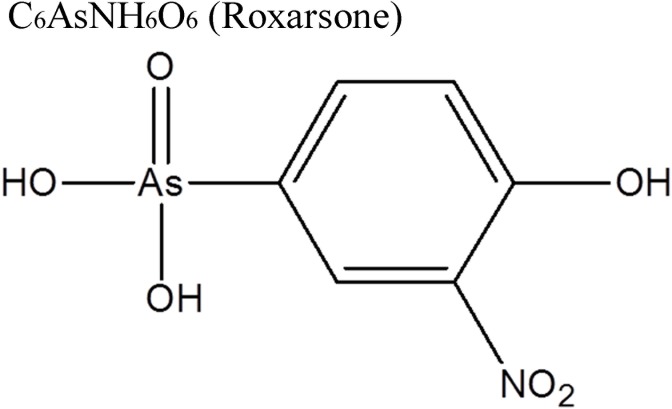
Schematic diagram and chemical formula of roxarsone.

Roxarsone (the schematic diagram and chemical formula seeing in Fig 1) has been widely used for decades in animal husbandry as a feed additive for controlling parasites and for growth promotion and is usually excreted unchanged in fresh manure [1–8]. The application of roxarsone in the poultry industry has been banned in most developed countries, while it is still heavily used in China as a feed additive and/or anti-coccidiosis agent [9]. Roxarsone itself is a moderately toxic compound, but it can easily and rapidly convert into more toxic products upon exposure (mainly direct release) to the environment or during the composting process (typically for organic fertilizer) of animal manure, resulting in severe environmental risks [10–13]. In nature, some of the most commonly detected (typically in contaminated soils and plants) transformation products of roxarsone include As(III), As(V), dimethylarsinic acid (DMA), monomethylarsonic acid (MMA) and 3-amino-4-hydroxybenzene arsonic acid (AHBAA) [5,11–14].

The redox chemistry of arsenic is crucial for its geochemical cycling, governing the chemical form, toxicity, bioavailability and mobility of arsenic in nature. Studies have shown the essential functions of ferric iron minerals in the environmental biogeochemistry of arsenic [15–19]. In nature, roxarsone can be adsorbed onto iron oxides, such as goethite and magnetite [17,19], forming immobilized arsenic compounds. Soluble Fe(II), which typically forms following reduction of iron oxide and Fe-bearing minerals by dissimilatory metal-reducing bacteria, may act as an efficient reducing agent in a variety of abiotic redox processes of arsenic [4,18,20]. Microorganisms were also found to play important functions in the biotransformation process of roxarsone [5,7,8,11]. For example, a pure culture of a *Clostridium* strain was able to anaerobically transform roxarsone to AHBAA [5]. *Shewanella oneidensis* MR-1, a well-known strain due to its capacity for respiration on a wide range of electron acceptors, is known to play important roles in the biogeochemical cycling of metals, metalloids, and radionuclides [21–25], facilitating metal mineralization, thereby creating an opportunity for enhanced arsenic adsorption [16,20,26,27].

Although the critical functions of microbial activity and iron (oxy) hydroxides in the fate of roxarsone in nature are well recognized, mechanistic understanding of the underlying biogeochemical process of roxarsone transformation remains unclear [7–9,28]. We studied roxarsone transformation dynamics in a model aqueous system and quantified how the presence of dissolved Fe(III), which associates with the metal-reducing microbial strain *S*. *oneidensis* MR-1, influences roxarsone transformation and affects its geochemical cycling.

## Materials and Methods

### Microbial Culture

*S*. *oneidensis* MR-1 (MCCC 1A01706) was cultivated anaerobically in Luria-Bertani (LB) broth at 30°C without shaking. Inoculum culture was harvested at the mid-log phase by centrifugation (5810R, Eppendorf, Hamburg, Germany) at 9000×g for 10 minutes (washed three times with the experimental medium, sterile basal medium, BM, for details see Tables A-C in S1 File), and was then re-suspended in BM for experiments. The experimental medium BM was buffered with 50.0 mmol/L bicarbonate according to Campbell et al. [18].

### *S*. *oneidensis* MR-1 Induced Roxarsone Reduction

Roxarsone reduction experiments were conducted anaerobically in butyl-stopper glass bottles (250 mL) at room temperature without shaking, at an initial microbial cell density of 8.0 × 10^6^ cells/mL (if not specified, identical experimental conditions were applied throughout the study). The initial roxarsone concentration of 1.00 mmol/L was applied, and 50.0 mmol/L sodium lactate was added as an exogenous carbon source (if not specified, identical sodium lactate was applied throughout the study). Nitrogen gas was purged into the butyl-stopper glass bottles for 15 minutes to remove oxygen. For the control tests, no exogenous carbon source (0 mmol/L of sodium lactate) was applied. The reference tests were conducted in the absence of both MR-1 inoculum and sodium lactate, with all other conditions identical. To study the effect of roxarsone concentration on microbial-induced roxarsone reduction, initial roxarsone concentrations of 0, 0.10, 0.50, 1.00 and 2.50 mmol/L (with the values determined according to the literature [29,30]) were used for batch experiments, respectively. All the experiments were carried out in triplicate in anoxic chamber (and for all experiments throughout the study if not specified).

### Effect of Fe(III) on Roxarsone Biotransformation

To investigate the functional roles of dissolved Fe(III) on roxarsone transformation in the presence of the MR-1 strain, the batch experiments were anaerobically carried out in 250 mL butyl-stopper glass bottles, with an initial microbial inoculum of 8.0 × 10^6^ cells/mL and 1.00 mmol/L roxarsone and 10.0 mmol/L Fe(III)-citrate. Initially, 50.0 mmol/L of sodium lactate was applied as an exogenous carbon source. For comparison, roxarsone reduction experiments were conducted in the absence of the MR-1 strain and Fe(III)-citrate, respectively, and with all other conditions identical. The reference experiments were conducted in the absence of both the MR-1 strain and Fe(III)-citrate, and all other conditions were identical.

Abiotic roxarsone reduction experiments were conducted anaerobically in 250 mL butyl-stopper glass bottles with an initial roxarsone concentration of 1.00 mmol/L and Fe(II) concentration of 5.0 mmol/L. Fe(II) solution was prepared according to Hohmann et al. [20].

### Analysis Methods

Four milliliter samples were taken from the experimental system at each time point for analysis. Microbial population density (biomass) was analyzed using optical density measurements at 600 nm. The measurement was calibrated against direct fluorescent microscopic cell counting on 0.2-*μ*m black polycarbonate filters (Waterman), with cells stained with a fluorescent dye (LIVE/DEAD *Bac*Light Bacterial Viability Kit, Invitrogen), and observed on an Olympus IX73 microscope [8].

The harvested aqueous samples were filtered through a 0.2-*μ*m syringe filter unit prior to determination of dissolved concentrations of Fe(II), total Fe, roxarsone and AHBAA. Fe(II) concentration was measured by a spectrophotometer using the ferrozine assay [31]. Roxarsone and AHBAA concentrations were quantified using an Agilent 1260 Infinity HPLC system equipped with an Agilent TC-C18(2) column (25 cm x 4.6 mm id, 5 μmol/L) according to Cortinas et al. [4] and were detected at 450 and 300 nm, respectively, with phosphate buffer (10 mmol/L, pH 7.2) applied as the mobile phase. Inorganic arsenic species, including As(III) and As(V), were estimated using LC-HG-AFS according to Huang et al. [12].

For transmission electron microscopy (TEM) analysis, the precipitate samples were harvested using a microcentrifuge at 9000×g (rinsed twice with anoxic deionized water) and were then anaerobically dried on carbon-coated copper grids prior to imaging. The images of the whole mounts were taken at 200 kV using a JEM-2100F TEM (JEOL, Japan) equipped with an energy-dispersive X-ray spectrometer (EDS) system. X-ray diffraction (XRD) analysis was performed on a Philips X'Pert Pro MPD system with Cu K*α* radiation (*λ* = 1.5418 Å) scanned between 10° and 70° (of 2*θ*).

Roxarsone, AHBAA and inorganic arsenic in the precipitates were extracted according to Liang et al. [8] with modification. The precipitate samples were collected at the end of the experiment by centrifugation (5810R, Eppendorf, Hamburg, Germany) at 10,000 rpm for 5 minutes and were then thoroughly mixed with a 200.0 mL mixture of 0.1 mmol/L H_3_PO_4_ and 0.1 mmol/L NaH_2_PO_4_·2H_2_O (1:9, v/v). The mixed solution samples were kept in a water bath at 55 ± 0.5°C for 10 h, followed by sonication for 10 minutes. The supernatants were collected and then filtered through a 0.22-*μ*m mixed cellulose membrane, and the filtrate was stored at 4°C for analysis.

## Results and Discussion

### Roxarsone Transformation in the Presence of *S*. *oneidensis* MR-1

**Fig 2:**
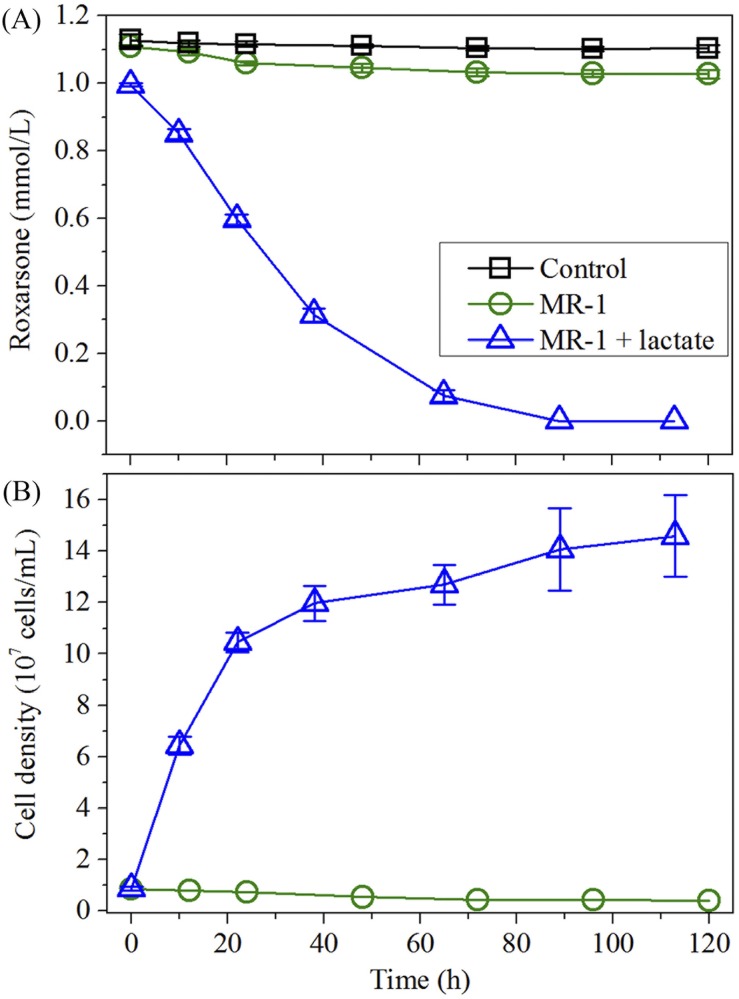
Roxarsone concentration profiles and microbial population growth (expressed as cell density) during the incubation time. (A) The average roxarsone concentration (mean ± s.d., n = 3) in the presence of *S*. *oneidensis* MR-1 and (or absence of) exogenous carbon source (lactate), and comparison to the control groups (absence of both MR-1 strain and lactate); and (B) microbial population growth.

To study the capability of *S*. *oneidensis* MR-1 to transform roxarsone, we performed roxarsone reduction experiments in the presence and absence of an exogenous carbon source of lactate, respectively, with inoculum of *S*. *oneidensis* MR-1, and with results shown in Fig 2. As expected, roxarsone concentration remained nearly unchanged throughout the incubation time for the control scenario, i.e., experiment in the absence of MR-1 and lactate [Fig 2A]. Addition of the MR-1 in the absence of lactate had little influence on roxarsone reduction, as observed in Fig 2A, which shows a barely reduced roxarsone concentration from 1.11 ± 0.002 to 1.03 ± 0.01 mmol/L after 120 h incubation time. Interestingly, the population of *S*. *oneidensis* MR-1 was found to gradually decline by half during the incubation time [Fig 2B]. The results revealed that *S*. *oneidensis* MR-1 was unable to efficiently reduce roxarsone in the absence of an exogenous carbon source, and the limited amount of reduced roxarsone was likely attributed to the endogenous respiration of MR-1 population. Roxarsone reduction was promoted following the introduction of lactate (50.0 mmol/L) into the experimental system, as shown in Fig 2A. For example, the estimated roxarsone concentration decreased rapidly from 1.00 ± 0.004 mmol/L to an undetectable level within 96 h, along with a quickly propagating microbial population that increased from 0.86 × 10^7^ to 14.59 × 10^7^ cells/L [Fig 2B]. The complete roxarsone transformation in the presence of an exogenous carbon source of lactate indicated that the MR-1 strain may use roxarsone as a terminal electron acceptor for anaerobic respiration, consistent with available observations where electron-donating substrates were found to stimulate roxarsone biotransformation into AHBAA [4].

### Effect of Initial Roxarsone Concentration on Its Biotransformation

**Fig 3:**
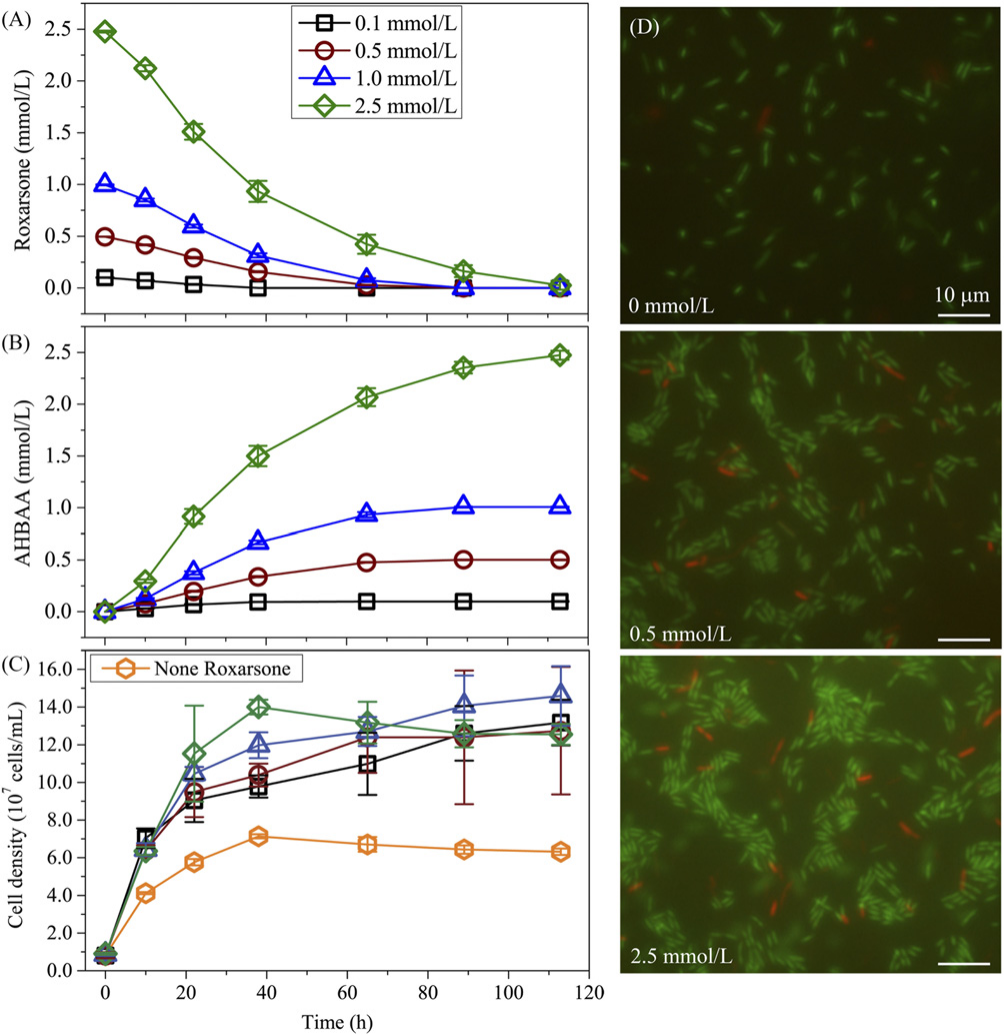
The effect of initial roxarsone concentration on *S*. *oneidensis* MR-1-induced roxarsone reduction. (A) The average roxarsone concentration (mean ± s.d., n = 3, also used for AHBAA and cell density values) profiles over time; (B) the concentration profiles of the transformation product AHBAA; (C) microbial population (expressed as cell density) growth over the incubation time course; and (D) fluorescent microscopy images of the experimental mixture samples harvested at 32 h after incubation with varying initial roxarsone concentrations of 0, 0.50 and 2.50 mmol/L as examples (green and red rods mark live and dead cells, respectively, white bar represents 10 *μ*m).

To quantify the effect of the initial roxarsone dose on its biotransformation in an anaerobic aqueous system, batch experiments were conducted with various initial roxarsone concentrations in the presence of *S*. *oneidensis* MR-1, with the results shown in Fig 3. For all scenarios, with the initial roxarsone concentration ranging from 0.10 to 2.50 mmol/L, roxarsone concentrations decreased gradually to nearly 0 within 113 h incubation. Interestingly, similar roxarsone reduction patterns were found for all concentrations, as observed in Fig 3A, indicating that there is little influence of initial roxarsone concentration on *S*. *oneidensis* MR-1-induced roxarsone biotransformation. Accordingly, AHBAA concentration continuously increased from 0 to up to 0.10 ± 0.003, 0.50 ± 0.003, 1.01 ± 0.004 and 2.47 ± 0.04 mmol/L for initial roxarsone concentrations of 0.10, 0.50, 1.00 and 2.50 mmol/L, respectively, with 113 h of incubation, showing a clear concurrence of its concentration and roxarsone reduction. It indicated that AHBAA was the only product of *S*. *oneidensis* MR-1-induced anaerobic roxarsone transformation, consistent with available observations where inorganic arsenic compounds were found only after the oxidation of the benzene ring of roxarsone [5]. The growth patterns of *S*. *oneidensis* MR-1 at various initial roxarsone levels were illustrated in Fig 3C, showing rapid microbial population growth for all scenarios, with obviously larger populations (expressed as cell density) for higher initial roxarsone concentrations. For example, microbial population density increased from 0.8 × 10^7^ to (7.1 ± 0.1) × 10^7^, (9.8 ± 0.6) × 10^7^, (10.4 ± 0.6) × 10^7^, (12.0 ± 0.7) × 10^7^, and (14.0 ± 0.4) × 10^7^ cells/mL at 38 h for 0, 0.10, 0.50, 1.00, and 2.50 mmol/L of initial roxarsone, respectively. It was also evidenced by fluorescent microscopy images of *S*. *oneidensis* MR-1 (with 0, 0.50 and 2.50 mmol/L initial roxarsone concentrations for examples) taken at 32 h after incubation, where significantly enlarged active cell populations were observed at higher initial roxarsone concentrations, while the population of dead cells barely changed, as shown in Fig 3D. Following that, microbial population growth slowed down or even decayed slightly until the end of the incubation [Fig 3D], which was likely due to the depletion of growth nutrients and resources. The results revealed that the initial roxarsone concentration did not inhibit, but rather stimulated population growth of *S*. *oneidensis* MR-1, e.g., there was nearly doubled cell density at 2.50 mmol/L roxarsone compared with that of 0 mmol/L of initial roxarsone (and with addition of 50.0 mmol/L lactate as the exogenous carbon source). *S*. *oneidensis* MR-1 is a well-known model bacterium able to reduce heavy metals [21, 32–34], and genome sequence analysis has predicted its capability for reducing nitro group of aromatic compounds [35–37]. In addition, Stolz and co-workers [5] simulated, based on density function theory, the electronic structure of roxarsone and concluded that the nitro group is the first to accept reducing equivalents for transformation, lending theoretical basis for the potential pathway of roxarsone biotransformation. Nevertheless, our results provided the first evidence of *S*. *oneidensis* MR-1 mediated direct roxarsone biotransformation in anaerobic aqueous systems.

### Effect of Fe(III) on Microbial-Induced Roxarsone Transformation

**Fig 4:**
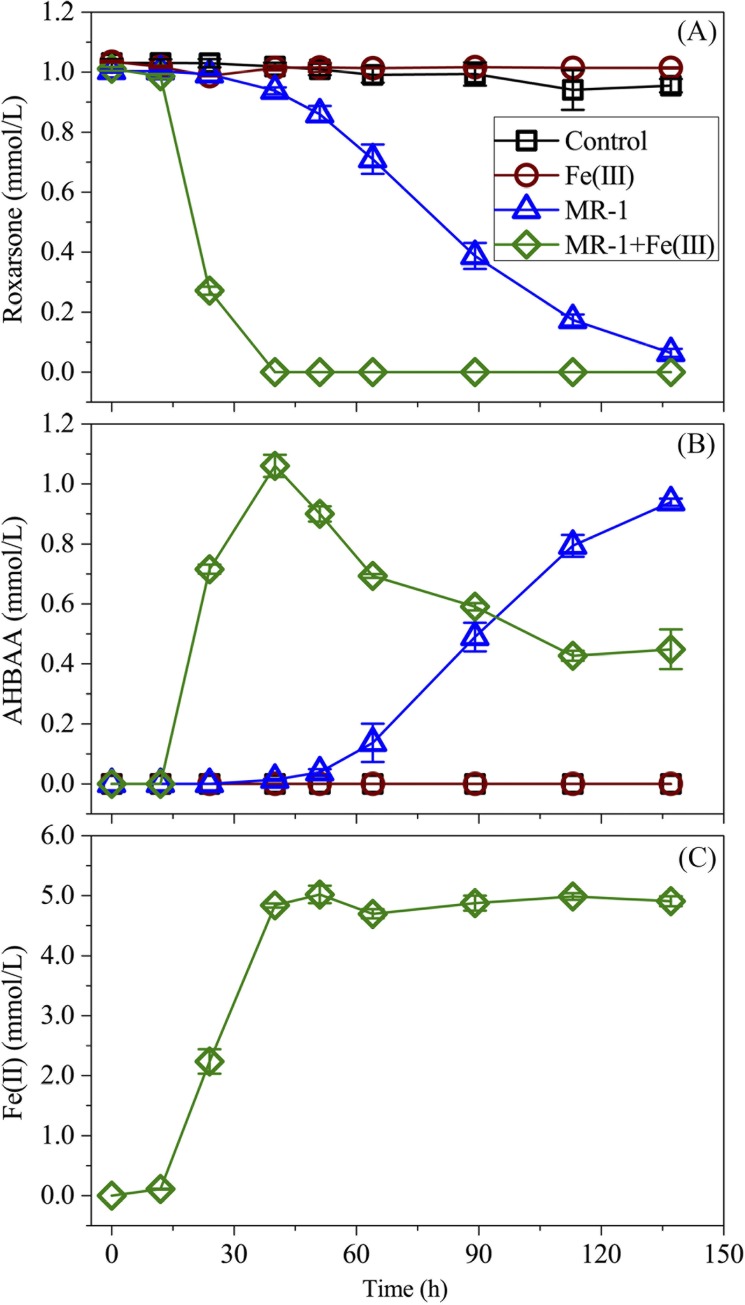
Roxarsone, AHBAA and/or Fe(II) concentration profiles for abiotic and microbial-associated roxarsone reduction. (A) The average roxarsone concentration (mean ± s.d., n = 3, and the same for AHBAA and Fe(II) concentration values) profiles over time in the presence of dissolved Fe(III), MR-1, Fe(III) plus MR-1, and no Fe(III) and MR-1, respectively; (B) AHBAA concentration profiles; and (C) aqueous Fe(II) concentration profile with the MR-1 strain and Fe(III).

**Fig 5:**
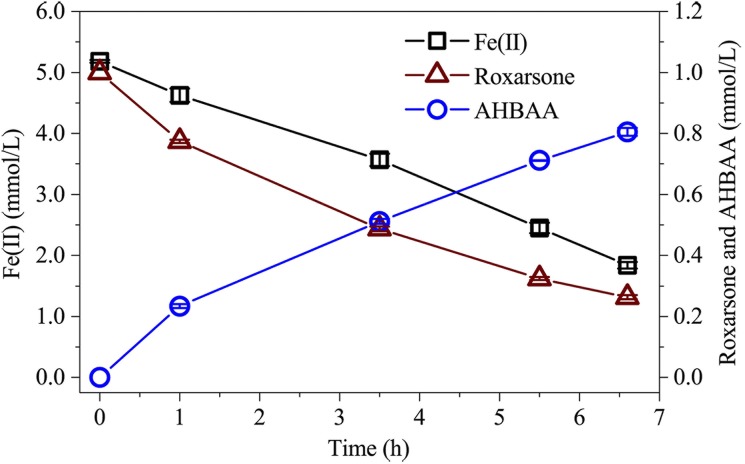
Roxarsone, AHBAA and Fe(II) concentration profiles for Fe(II)-induced roxarsone reduction. The average concentration (mean ± s.d., n = 3) of roxarsone, AHBAA and aqueous Fe(II) for the scenario of Fe(II)-induced roxarsone reduction.

In the absence of MR-1 inoculum and Fe(III), no obvious roxarsone reduction was observed, as shown in Fig 4A of nearly stable roxarsone concentration throughout the incubation time of 138 h. Accordingly, no obvious AHBAA was detected during the incubation period [Fig 4B]. Introduction of Fe(III) in the absence of *S*. *oneidensis* MR-1 did not result in roxarsone reduction, as observed in Fig 4A and 4B, where the roxarsone and AHBAA concentrations were found to be nearly unchanged. In contrast, with the introduction of Fe(III) and *S*. *oneidensis* MR-1, roxarsone concentration was observed to drop (at approximately 12 h after incubation) from 0.99 ± 0.01 mmol/L to an undetectable level at 40 h after incubation, following a sluggish decrease at the beginning of incubation that was most likely related to the population growth delay of *S*. *oneidensis* MR-1 [Fig 4A]. As a comparison, a gradually decreased roxarsone concentration pattern (from 1.00 to 0.06 mmol/L at 138 h after incubation) was found for the scenario without Fe(III) addition [Fig 4A]. The results revealed that Fe(III) ions stimulated roxarsone transformation. Accordingly, the Fe(II) concentration was found to increase (albeit slightly delayed) with decreasing roxarsone concentration and nearly leveled out upon complete reduction of roxarsone at 40 h after incubation [Fig 4C]. The results indicated that *S*. *oneidensis* MR-1-yielded Fe(II) promoted roxarsone transformation. Indeed, direct chemical reduction of roxarsone in the presence of the chemical reducing reagent Fe(II) was reported [4]. In a separate experiment, we also observed direct transformation of roxarsone into AHBAA in an anaerobic aqueous environment in the presence of dissolved Fe(II), while the absence of *S*. *oneidensis* MR-1, as observed in Fig 5, simultaneously decreased roxarsone and Fe(II) concentrations that were associated with the symmetrically increased AHBAA concentration. The results revealed that it was microbially induced Fe(III) transformation (into Fe(II)) that facilitated roxarsone reduction. However, the accumulation of Fe(II) (albeit slightly delayed as compared to that of roxarsone reduction) prior to complete roxarsone reduction indicated that the MR-1-induced Fe(III) transformation process was faster than Fe(II)-induced roxarsone transformation. Considering the well-known ability of metal metabolism for *S*. *oneidensis* MR-1 [22,33,34,38] and the initial addition of 10.0 mmol/L of Fe(III) and the approximately 4.9 mmol/L final yield of Fe(II), there was at least (not considering regenerated Fe(II) by *S*. *oneidensis* MR-1) 5.1 mmol/L of *S*. *oneidensis* MR-1-yielded Fe(II) contributed to roxarsone transformation. Taking into account Fe(II) and roxarsone reaction stoichiometry [4] that the complete transformation of 1.0 mmol/L roxarsone (to AHBAA) requires 6.0 mmol/L of Fe(II), 5.1 mmol/L Fe(II) could yield 0.85 mmol/L of roxarsone transformation. Although the regenerated Fe(II) by *S*. *oneidensis* MR-1 [4] may still contribute to the anaerobic transformation of the rest of the 0.15 mmol/L roxarsone, there was also a possibility that this portion of roxarsone would (partially) rely on direct transformation by *S*. *oneidensis* MR-1. AHBAA was detected as the sole transformation product, with its concentration accumulating rapidly, after a 12 h delay. It increased to 1.06 ± 0.04 mmol/L within 40 h after incubation for the scenario with Fe(III) addition and to 0.94 ± 0.01 mmol/L (with a relatively gentle increase) at 138 h after incubation without Fe(III) addition [Fig 4B].

**Fig 6:**
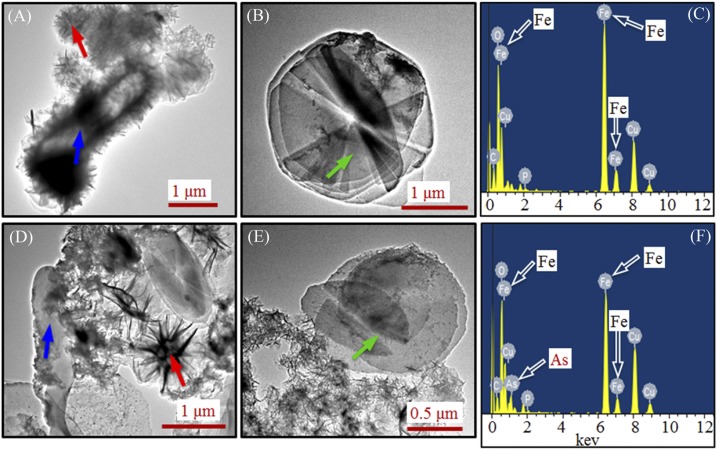
The TEM images and EDS analysis results of the precipitate samples harvested at 80 h after incubation. (A) and (B) TEM images of precipitate samples for the scenario with only Fe(III) and *S*. *oneidensis* MR-1 and no roxarsone; (C) EDS analysis results of the entire area of the sample (A); (D) and (E) TEM images in the presence of roxarsone, Fe(III) and *S*. *oneidensis* MR-1; and (F) EDS results of the entire area of the sample (D). Blue arrows in panels A and D mark microbial cells partially covered with precipitates, red (A and D) and green (B and E) arrows mark two different morphologies of the formed precipitates, and open arrows in panels C and F mark Fe and As elements for EDS analysis.

**Fig 7:**
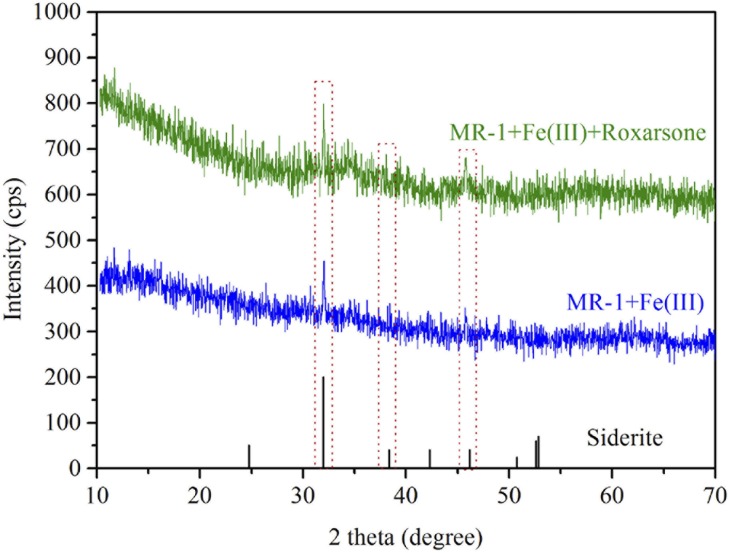
X-ray diffraction analysis patterns for the precipitate samples. X-ray diffraction analysis patterns for the same precipitate samples of Fig 6A [MR-1+Fe(III)] and 6D [MR-1+Fe(III)+roxarsone] and comparison with the characteristic patterns of siderite. The dashed squares mark the typical characteristic XRD patterns of siderite.

Interestingly, there was a gradual (yet significant) decay of AHBAA concentration following its peak value of 1.06 ± 0.04 mmol/L (that occurred at 40 h after incubation) to 0.45 ± 0.07 mmol/L at the end of experiment (i.e., following the complete roxarsone reduction), which was associated with clear precipitate formation and was found only in the scenario with Fe(III) addition [Fig 4B]. The results also suggested that an additional mechanism must be responsible for the nearly 55% drop in AHBAA concentration. Additional analysis (for details see Analysis Methods) of the precipitates accounted for 0.56 ± 0.02 mmol/L of AHBAA, indicating that AHBAA was the only product of roxarsone transformation. It was recently determined that formation of secondary Fe(II) and Fe(II)/Fe(III) minerals may lead to immobilization of dissoluble inorganic arsenic compounds [18,23,25]. Following this line, we performed TEM and EDS analysis of the precipitate samples harvested at 80 h after incubation, with the results shown in Fig 6. The TEM images showed different types of minerals (e.g., dish-like and stellate fiber-like) formed following microbial-induced Fe(III) reduction both without [Fig 6A and 6B] and with roxarsone [Fig 6D and 6E], and with Fe(III) and the MR-1 strain. As expected, both Fe and As were detected from the precipitate samples harvested from the treatments with roxarsone [Fig 6F], but there was no As (while Fe was present) for that with no roxarsone [Fig 6C]. A separate XRD analysis showed identical XRD patterns of the yielded minerals (precipitates) with that of siderite (FeCO_3_) (Fig 7), indicating secondary iron mineralization which was likely attributed to the presence of 50.0 mmol/L bicarbonate (that serves as the buffering agent). These patterns are also reminiscent of the secondary iron minerals observed in other microbial-induced reductions of Fe(III) and/or inorganic arsenic [15,23,25,27,34] and thus lend support to secondary Fe(II) and Fe(II)/Fe(III) mineralization along with *S*. *oneidensis* MR-1-induced roxarsone and Fe(III) reductions that eventually led to the subsequent immobilization of the otherwise dissoluble AHBAA [23,39].

Interest in natural biochemical functions as key factors for untangling the environmental dynamics of arsenic pollutants has yielded significant advances [7,8,15,25,27,34], yet mechanistic understanding of the biotransformation processes of the widely spread roxarsone contaminant remains elusive due to limited experimental information. This study provided the first evidence that *S*. *oneidensis* MR-1, a common metal-reducing bacterium, initiates roxarsone transformation. The results have shown that *S*. *oneidensis* MR-1 was able to directly transform roxarsone into AHBAA in anaerobic aqueous systems, and the presence of dissolved Fe(III) significantly enhanced roxarsone reduction owning to *S*. *oneidensis* MR-1-induced Fe(III) transformation, whereby the yielded Fe(II) acted as an efficient chemical reagent for roxarsone reduction [4]. In addition, the subsequent secondary iron mineralization created conditions for adsorption of organic arsenic transformation products to the Fe(II) and Fe(II)/Fe(III) minerals and thereby led to organoarsenic immobilization. Considering the widely distributed metal-reducing bacteria and rich presence of iron oxides, carbonate and phosphate in nature [33,34,37,39–41], such biogeochemical processes are expected to occur extensively in natural systems and would play a critical role in the transformation dynamics of roxarsone in nature, typically in anoxic saturated soils, e.g., rice paddies, and aquifers. With the quantitative estimations, this study provides new insights into the mechanistic understanding of the environmental dynamics of roxarsone, which are important for successful risk assessment.

## Supporting Information

S1 File Table A. The composition of the bacterial minimal medium (BM); Table B. The composition of the vitamin mixture used in bacterial minimal medium; and Table C. The composition of the mineral mixture used in bacterial minimal medium.
(PDF)
