# Supplementary material for: Shewanella oneidensis MR-1-Induced Fe(III) Reduction Facilitates Roxarsone Transformation
Source: PLoS One. 2016 Apr 21;11(4):e0154017. doi: 10.1371/journal.pone.0154017 (PMC4839622; doi:10.1371/journal.pone.0154017)
Supplement: S1 File — Table A. The composition of the bacterial minimal medium (BM); Table B. The composition of the vitamin mixture used in bacterial minimal medium; and Table C. The composition of the mineral mixture used in bacterial minimal medium. (PDF) [file pone.0154017.s001.pdf]

Supporting Information

Shewanella Oneidensis MR-1-Induced Fe(III) Reduction Facilitates Roxarsone Transformation

Running Title: Microbial Facilitated Roxarsone Transformation

Guowei Chen[1], Zhengchen Ke[1], Tengfang Liang[1], Li Liu[1,*], Gang Wang[2,*]

[1] School of Civil and Hydraulic Engineering, Hefei University of Technology, Hefei 230009, China

[2] Department of Soil and Water Sciences, China Agricultural University, Beijing 100193, China

* Corresponding author

E-mail: gangwang@cau.edu.cn (GW) or lliu@hfut.edu.cn (LL)

Table A. The composition of the bacterial minimal medium (BM).

Chemical	Concentration
K_2HPO_4	50.0 mg/L
KH_2PO_4	35.0 mg/L
NaCl	460.0 mg/L
$(NH_4)_2SO_4$	225.0 mg/L
$MgSO_4 \cdot 7H_2O$	117.0 mg/L
$NaHCO_3$	50.0 mmol/L
$NaC_3H_5O_3$	50.0 mmol/L
Vitamin Mix[1]	5.0 mL
Mineral Mix[2]	5.0 mL

[1,2] Details in Tables B and C.

Table B. The composition of vitamin mix solution used in bacterial minimal medium.

Constituent	Concentration
Biotin	2.0 mg/L
Folic acid	2.0 mg/L
Pyridoxine HCl	10.0 mg/L
Riboflavin	5.0 mg/L
Thiamine	5.0 mg/L
Nicotinic acid	5.0 mg/L
Pantothenic acid	5.0 mg/L
B-12	0.1 mg/L
p-aminobenzoic acid	5.0 mg/L
Thioctic acid	5.0 mg/L

Table C. The composition of minerals mix solution used in bacterial minimal medium.

Constituent	Concentration
NTA	1500.0 mg/L
$MgSO_4$	3000.0 mg/L
NaCl	1000.0 mg/L
$MnSO_4 \cdot H_2O$	500.0 mg/L
$FeSO_4 \cdot 7H_2O$	100.0 mg/L
$CaCl_2 \cdot 2H_2O$	100.0 mg/L
$CoCl_2 \cdot 6H_2O$	100.0 mg/L
$ZnCl_2$	130.0 mg/L
$CuSO_4 \cdot 5H_2O$	10.0 mg/L
$AlK(SO_4) \cdot 12H_2O$	10.0 mg/L
H_3BO_3	10.0 mg/L
Na_2MoO_4	25.0 mg/L
$NiCl_2 \cdot 6H_2O$	24.0 mg/L
$Na_2WO_4 \cdot 2H_2O$	25.0 mg/L
